#  Evolutionary Dynamics of rDNA Clusters in Chromosomes of Five Clam Species Belonging to the Family Veneridae (Mollusca, Bivalvia)

**DOI:** 10.1155/2014/754012

**Published:** 2014-05-22

**Authors:** Concepción Pérez-García, Ninoska S. Hurtado, Paloma Morán, Juan J. Pasantes

**Affiliations:** ^1^Departamento de Bioquímica Xenética e Inmunoloxía, Universidade de Vigo, 36310 Vigo, Spain; ^2^Department of Biogeochemistry and Ecotoxicology, Laboratory of Ecotoxicology, rue de l'Ile d'Yeu, BP 21105, 44311 Nantes Cedex 03, France

## Abstract

The chromosomal changes accompanying bivalve evolution are an area about which few reports have been published. To improve our understanding on chromosome evolution in Veneridae, ribosomal RNA gene clusters were mapped by fluorescent* in situ* hybridization (FISH) to chromosomes of five species of venerid clams (*Venerupis corrugata*,* Ruditapes philippinarum*,* Ruditapes decussatus*,* Dosinia exoleta*, and* Venus verrucosa*). The results were anchored to the most comprehensive molecular phylogenetic tree currently available for Veneridae. While a single major rDNA cluster was found in each of the five species, the number of 5S rDNA clusters showed high interspecies variation. Major rDNA was either subterminal to the short arms or intercalary to the long arms of metacentric or submetacentric chromosomes, whereas minor rDNA signals showed higher variability. Major and minor rDNAs map to different chromosome pairs in all species, but in* R. decussatus* one of the minor rDNA gene clusters and the major rDNA cluster were located in the same position on a single chromosome pair. This interspersion of both sequences was confirmed by fiber FISH. Telomeric signals appeared at both ends of every chromosome in all species. FISH mapping data are discussed in relation to the molecular phylogenetic trees currently available for Veneridae.

## 1. Introduction

The nuclear genes that code for the ribosomal RNA in eukaryotes are organized into two different multigene families. Major rDNA repeats consist of a transcriptional unit which codes for the 18S, 5.8S, and 28S rRNAs, separated by two internal transcribed spacers (ITS1 and ITS2) and an intergenic spacer (IGS). Minor rDNA repeats consist of a sequence which codes for the 5S rRNA and a nontranscribed spacer (NTS). Although in most eukaryotes in which the organization of these genes is known the two types of rDNAs are located on different chromosome pairs, an increasing number of studies have reported the existence of clusters of both major and minor rRNA genes mapping to the same chromosomal position in many species [1, 2]. Furthermore, the changes in both number and chromosomal position of the rDNA clusters have helped to solve some uncertainties in the phylogenies of some groups of organisms [3, 4].

The family Veneridae (Bivalvia, Heterodonta) includes more than 800 living species distributed worldwide which have adapted to a wide range of marine environments [5, 6]. The phylogenetic relationships among the species of this family have been investigated using both morphological and molecular features [6–9], but, in terms of characterizing their chromosomes, current knowledge is limited to mitotic chromosome numbers and karyotypes of just a few species [10–13], a restriction endonuclease banding pattern in* Ruditapes decussatus* [14], and the location of telomeric, rDNA and/or core histone gene sequences in* Mercenaria mercenaria* [15, 16],* Dosinia exoleta* [13],* R. decussatus *and* R. philippinarum* [17],* Polititapes* (*Venerupis) aureus, *and* P. (Tapes) rhomboides* [18].

In order to advance the chromosomes characterization of bivalve species and gain some insights on their evolution, we hybridized major rDNA, 5S rDNA, and telomeric probes to mitotic chromosomes and surface spread synaptonemal complexes of five species of clams of different subfamilies of the family Veneridae:* Venerupis corrugata*,* Ruditapes philippinarum,* and* R. decussatus *(Tapetinae, clade A1 in [6]),* Dosinia exoleta* (Dosiniinae, clade A4 in [6]), and* Venus verrucosa* (Venerinae, clade A2 in [6]). The anchoring of the chromosomal mapping data obtained, together with previously published data for* Polititapes aureus* and* P. rhomboides* [18] (Tapetinae, clade A1 in [6]) and* Mercenaria mercenaria* [16] (Chioninae, clade A3 in [6]), to the phylogenetic tree proposed by Chen et al. [6] allows us to shed some light on the chromosomal changes that occurred during the evolution of this bivalve family.

## 2. Materials and Methods

### 2.1. Biological Material

Specimens of the pullet carpet shell* Venerupis corrugata *(*pullastra*) (Gmelin 1791), the grooved carpet shell* Ruditapes decussatus* (Linnaeus 1758), the Japanese carpet shell* Ruditapes philippinarum *(Adams and Reeve 1850), the rayed artemis* Dosinia exoleta *(Linnaeus 1758), and the warty venus* Venus verrucosa* (Linnaeus 1758) were collected from field and cultured populations in Ría de Pontevedra and Ría de Vigo (NW Spain). Identification of the species level was performed on the basis of the morphology of their shells and the degree of separation of their siphons. The nomenclature used for these taxa followed the World Register of Marine Species database (http://www.marinespecies.org/).

Male and female juvenile individuals were maintained in the laboratory in 10 L tanks of aerated, filtered, sea water at 18 ± 1°C. The animals were fed on a suspension of algal cells (*Isochrysis galbana*) for at least 10 days in order to promote both somatic growth and gonadic maturation. Mature specimens were also collected during the reproductive season.

### 2.2. Probe Preparation

Total genomic DNA was extracted from ethanol-preserved adductor muscles [19]. Approximately 3 mg of tissue was homogenized in hexadecyltrimethylammonium bromide (CTAB) buffer and digested overnight with pronase (1.5 mg/mL) at 60°C. DNA was extracted with chloroform/isoamyl alcohol (24/1) and stored at 4°C.

FISH probes were obtained by polymerase chain reaction (PCR) amplifications performed in 20 *μ*L of a solution containing 50 ng DNA, 1xPCR buffer, 50 *μ*M each dNTP, 2.5 mM MgCl_2_, 1 *μ*M each primer, and 1 U BIOTAQ DNA polymerase (Bioline). Universal primers were used to amplify the whole internal transcribed spacer (ITS) region of the major rDNA cluster (*ITS4*: 5′-TCCTCCGCTTATTGATATGC-3′,* ITS5*: 5′-GGAAGTAAAAGTCGTAACAAGG-3′ [20]). For the amplification of the whole repeat unit of the 5S rDNA, we designed primers (*5SD*: 5′-CAACGTGATATGGTCGTAGAC-3′,* 5SR*: 5′-AACACCGGTTCTCGTCCGATC-3′) from the sequence of the 5S rRNA of* Mytilus edulis* [21].

After 5 min of denaturation at 95°C, 30 cycles of amplification were performed (95°C, 30 s; 48°C, 30 s; 72°C, 30 s for the ITS, 50 s for the 5S rDNA). A final extension step of 7 min at 72°C was applied. All reactions were performed in a GeneAmp PCR system 9700 (Applied Biosystems), and PCR products were examined by electrophoresis on 2% agarose gels.

The amplified ITSs obtained from the five species and the 5S rDNA from* Venerupis corrugata* were labeled with biotin-16-dUTP (Roche Applied Science) and/or digoxigenin-11-dUTP (10x DIG Labeling Mix, Roche Applied Science) using a nick translation kit (Roche Applied Science). The remaining 5S rDNAs were directly labeled by PCR with either biotin-16-dUTP (20 *μ*M) or digoxigenin-11-dUTP (5 *μ*M).

### 2.3. Mitotic Chromosome, Synaptonemal Complex, and Release Chromatin Fiber Preparation

Mitotic chromosome preparations were obtained according to Pasantes et al. [22]. Juvenile specimens were housed in 0.5 L beakers and exposed to colchicine (0.005%) for 12 h. Gills were excised and immersed in 50% and 25% sea water for 1 h and fixed with ethanol/acetic acid for 1 h. Small pieces of tissue were dissociated in 60% acetic acid, and the cell suspension was dropped onto slides heated to 50°C.

Surface spreading of synaptonemal complexes (SCs) was performed as indicated by Hurtado and Pasantes [13]. Fresh male gonadic tissue was minced in 0.4 M NaCl and centrifuged several times to remove the sperm. One drop of the cellular suspension was spread for 1–5 min on clean slides covered by a spreading medium (0.1 M sucrose, 0.5% Triton X-100). The slides were then flooded with paraformaldehyde (4%) and the liquid mixture allowed settling overnight. After draining briefly, the slides were rinsed in distilled water and air-dried.

Chromatin fibers were released as described by Fidlerova et al. [23]. In brief, the cellular suspension obtained following the protocol for mitotic chromosome preparation was centrifuged for 10 min at 1200 rpm, and the supernatant was discarded. The pellet was resuspended in fresh fixative and dropped on clean slides. After leaving to evaporate briefly, slides were immersed in 1x PBS for 1 min and chromatin fibers were released with 0.05 M NaOH in 30% ethanol.

### 2.4. Fluorescent* In Situ* Hybridization (FISH)

FISH, SC-FISH, and Fiber-FISH experiments using biotin and digoxigenin labeled species-specific ITS and 5S rDNA probes were performed following the methods published elsewhere [13, 18]. Slides were pretreated with RNase and pepsin before denaturation. Preparations were denatured for 2 min at 70°C (mitotic chromosomes and released chromatin fibers) or 80°C (meiotic chromosomes). Hybridization was performed overnight at 37°C. Signal detection was carried out with fluorescein avidin and biotinylated anti-avidin for the biotinylated probes and with mouse anti-digoxigenin and anti-mouse TRITC for the probes labeled with digoxigenin. Slides were counterstained for 8 min with 4′-6-diamidino-2-phenylindole (DAPI: 0.14 *μ*g mL^−1^ in 2x SSC) and mounted in antifade (Vectashield, Vector). In addition, we also performed FISH using a vertebrate telomeric (C_3_TA_2_)_3_ peptide nucleic acid (PNA) probe (Applied Biosystems) following the protocol indicated by the supplier.

Slide visualization and photography were performed using a Nikon Eclipse-800 microscope equipped with an epifluorescence system. A minimum of 5 individuals per species and 20 metaphases per individual were recorded for each probe on mitotic chromosomes. For SC-FISH, a minimum of 5 individuals per species and 30 SC spreads per individual were recorded for each probe. Separated images for each fluorochrome were obtained with a DS-Qi1Mc CCD camera (Nikon) controlled by the NIS-Elements software (Nikon). The merging of the images was performed with Adobe Photoshop.

## 3. Results

**Figure 1:**
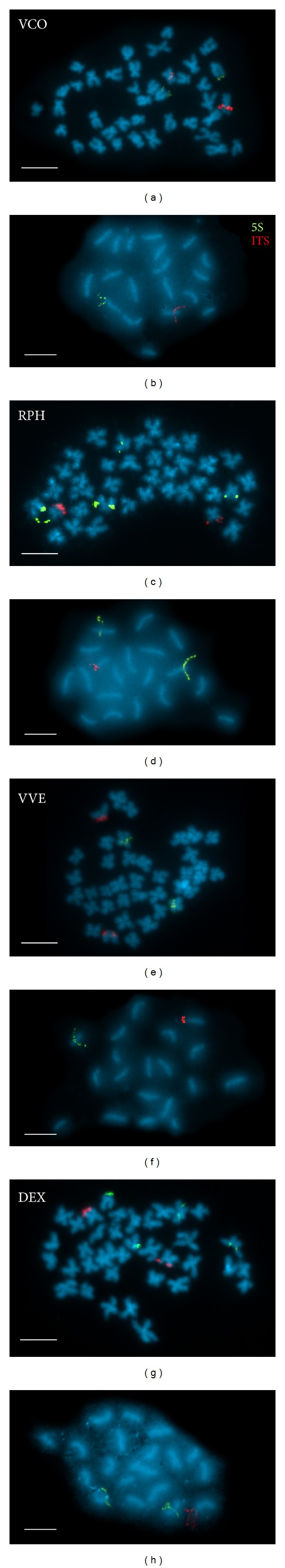
Chromosomal location of 5S rDNA and major rDNA to mitotic chromosomes ((a), (c), (e), and (g)) and surface spread synaptonemal complexes ((b), (d), (f), and (h)) of* Venerupis corrugata *(VCO, (a) and (b)),* Ruditapes philippinarum *(RPH, (c) and (d)),* Venus verrucosa *(VVE, (e) and (f)), and* Dosinia exoleta *(DEX, (g) and (h)) counterstained with DAPI. Major rDNA signals (ITS; digoxigenin, Rhodamine, red) are intercalary to the long arms of a pair of metacentric chromosomes (bivalents) in* Venerupis corrugata *((a) and (b)) but terminal to the short arms of a metacentric chromosome pair (bivalent) in* Ruditapes philippinarum *((c) and (d)) and* Dosinia exoleta *((g) and (h)) and a submetacentric chromosome pair (bivalent) in* Venus verrucosa *((e) and (f)). 5S rDNA signals (5S; biotin, fluorescein, green) are subterminally located on the short arms of a subtelocentric chromosome pair (bivalent) in* Venerupis corrugata *((a) and (b)), close to the centromere on the short arms of a metacentric chromosome pair (bivalent) in* Venus verrucosa *((e) and (f)), intercalary to the long arms of submetacentric and subtelocentric chromosome pairs (bivalents) in* Ruditapes philippinarum *((c) and (d)), terminal to the short arms, and centromeric on the long arms of two submetacentric chromosome pairs (bivalents) in* Dosinia exoleta *((g) and (h)). Note that all signals are on different chromosome pairs (bivalents). Scale bars: 5 *μ*m.

**Figure 2:**
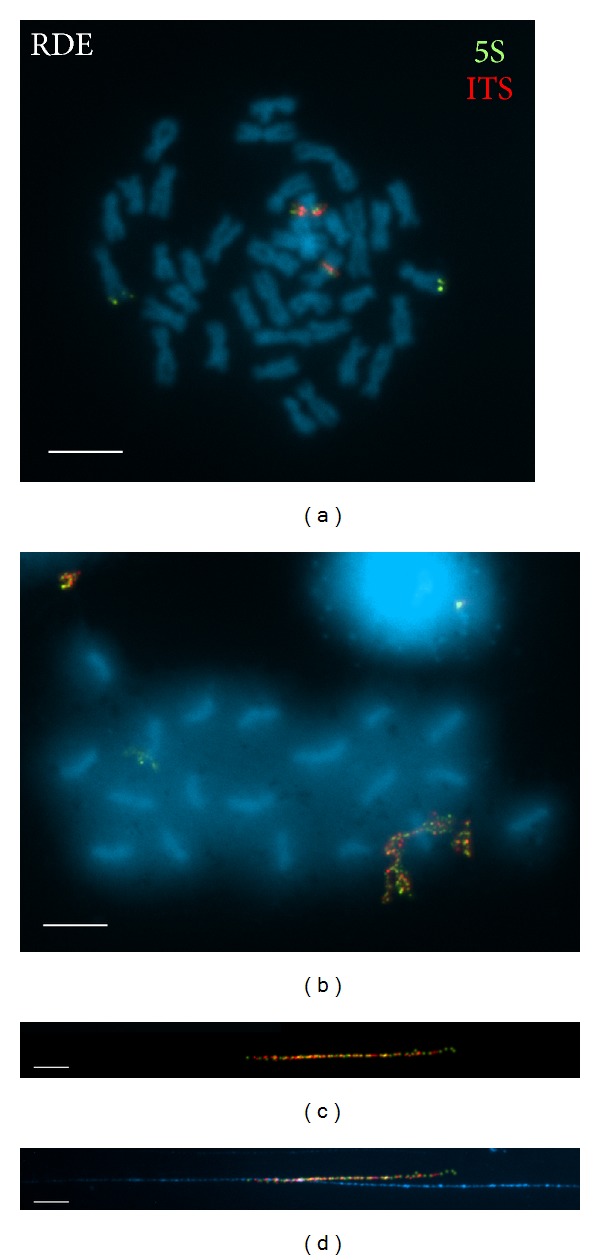
Two-color FISH of major rDNA (ITS, digoxigenin, Rhodamine, red) and 5S rDNA (5S, biotin, fluorescein, green) probes to mitotic chromosomes (a), surface spread synaptonemal complexes (b), and released chromatin fibers ((c) and (d)) of* Ruditapes decussatus* (RDE). Major rDNA red signals are intercalary on the long arms of a submetacentric chromosome pair (bivalent) overlapping 5S rDNA green signals, therefore giving yellow signals. The remaining 5S rDNA signals are terminal to the long arms of a subtelocentric chromosome pair (bivalent). The green and red signals that overlap in mitotic and meiotic chromosomes are clearly distinct on the released chromatin fibers and show an interspersion pattern ((c) and (d)). Scale bars: 5 *μ*m.

All specimens of the five clam species presented mitotic metaphase plates showing 38 chromosomes and meiotic prophase I plates with 19 bivalents (Figures 1 and 2).

Single- and double-color FISH experiments showed that major rDNAs mapped to a single locus in all five clam species (Figures 1 and 2). Intercalary signals appeared in mitotic metaphase chromosomes and meiotic prophase I bivalents in both* Venerupis corrugata* (Figures 1(a) and 1(b)) and* Ruditapes decussatus* (Figures 2(a) and 2(b)). The signals were located on the long arms of metacentric and submetacentric chromosome pairs, respectively. On the other hand, signals were subterminal to short arms in* R. philippinarum *(Figures 1(c) and 1(d)),* Venus verrucosa* (Figures 1(e) and 1(f)), and* Dosinia exoleta *(Figures 1(g) and 1(h)).

Minor rDNA signals mapped to a single locus in* Venerupis corrugata* and* Venus verrucosa* and to two loci on different chromosomes in* Ruditapes philippinarum*,* R. decussatus,* and* Dosinia exoleta*. The single 5S rDNA cluster was subterminal to the long arms of a subtelocentric chromosome in* V. corrugata* (Figures 1(a) and 1(b)) but almost centromeric on the short arms of a metacentric chromosome in* V. verrucosa* (Figures 1(e) and 1(f)). The two 5S rDNA clusters present in the remaining three species were subterminal and intercalary to the long arms of two subtelocentric chromosomes in* R. philippinarum* (Figures 1(c) and 1(d)), subterminal to the short arms and almost centromeric to the long arms of two submetacentric chromosomes in* D. exoleta* (Figures 1(g) and 1(h)), and intercalary and subterminal to the long arms of submetacentric and subtelocentric chromosomes in* R. decussatus* (Figures 2(a) and 2(b)).

Double-color FISH experiments showed that major and minor rDNAs mapped to different chromosome pairs in* Venerupis corrugata* (Figures 1(a) and 1(b)),* Ruditapes philippinarum *(Figures 1(c) and 1(d)),* Venus verrucosa* (Figures 1(e) and 1(f)), and* Dosinia exoleta *(Figures 1(g) and 1(h)). However, in* R. decussatus,* one of the minor rDNA gene clusters and the major rDNA cluster were located in the same positions on the same chromosome pair resulting in overlapping signals (Figures 2(a) and 2(b)). In order to determine whether these signals were really overlapping or close together but distinct, double-color FISH experiments were performed on extended chromatin fibers of* R. decussatus*. As clearly shown in Figures 2(c) and 2(d), all fibers showing major rDNA signals also presented signals corresponding to 5S rDNA. When the fibers lack enough extension, these red and green signals overlap, but when stretched, the signals were clearly interspersed. On the same slides, other fibers only showed 5S rDNA signals.

**Figure 3:**
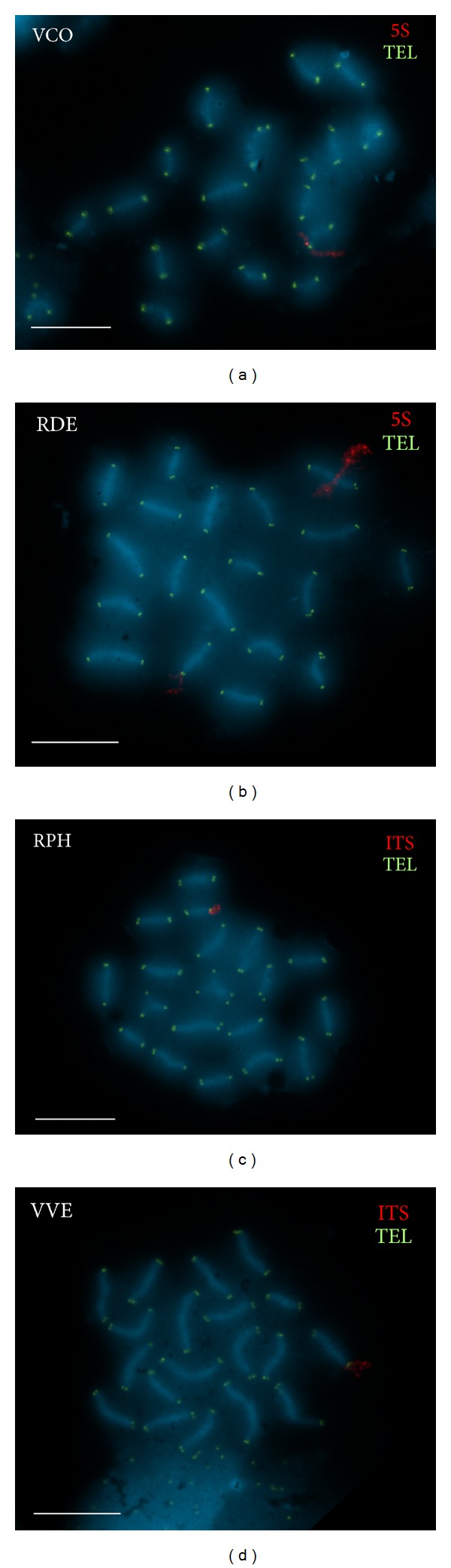
Two-color FISH of telomeric (TEL, fluorescein, green) and 5S or major rDNA (5S or ITS, digoxigenin, Rhodamine, red) probes to surface spread synaptonemal complexes of* Venerupis corrugata *(VCO (a)),* Ruditapes decussatus* (RDE (b)),* Ruditapes philippinarum *(RPH (c)), and* Venus verrucosa *(VVE (d)) counterstained with DAPI. Note that all telomeric signals appear as tightly packed single or double green dots attached to the SCs at both ends of each bivalent in the four clam species. On the other hand, the subterminal red signals corresponding to 5S (VCO (a); RDE (b)) and ITS (RPH (c), VVE (d)) rDNA probes surround the telomeric signals radiating away from the SCs in all clams. Scale bars: 5 *μ*m.

Sequential FISH experiments using a vertebrate telomeric (C_3_TA_2_)_3_ PNA probe followed by a second FISH using major rDNA or 5S rDNA probes showed terminal signals at the ends of the sister chromatids of every mitotic chromosome and of the SCs in every prophase I bivalent (Figure 3). No intercalary signals were detected. As previously reported in* Dosinia exoleta* [13], telomeric signals appeared to be quite condensed and tightly associated at the ends of the SCs in pachytene chromosomes. On the other hand, rDNA signal dots on SC spreads were always associated with chromatin that loops away from the SC.

**Figure 4:**
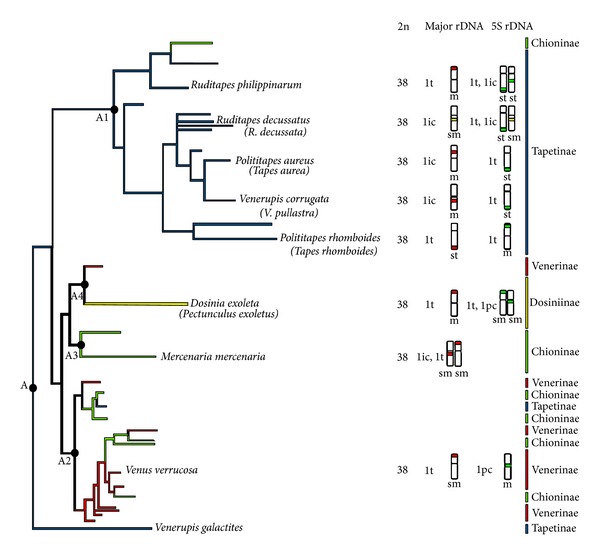
Schematic representation of the distribution of major (red) and 5S (green) rDNA clusters on chromosomes of Veneridae. Yellow marks indicate linkage of major and minor rDNAs. The species are organized according to the phylogenetic tree (clade A) proposed by Chen et al. [6] (left) and also assigned to subfamilies according to the accepted traditional classification (right).

Figure 4 shows a summary of the rDNA mapping results currently available for the family Veneridae. The species were ordered according to the phylogenetic tree proposed by Chen et al. [6] and assigned to both their clade groups (A1, A2, A3, and A4) and the traditional subfamily classification (Tapetinae, Chioninae, Venerinae, and Dosiniinae). 

## 4. Discussion

To date, karyological data are available for only a few species of the family Veneridae [10, 11, 24]. Both the chromosome numbers and the karyotypes determined in this work for* Ruditapes philippinarum*,* R. decussatus*,* Venerupis corrugata*,* Venus verrucosa, *and* Dosinia exoleta* are in agreement with previous results [12, 13, 25, 26] and further confirm that, unlike other families within the order Veneroida in which interspecific differences in chromosome numbers have been detected, all Veneridae species have the same chromosome number, 2*n* = 38. Nevertheless, the high variation in karyotype composition detected in these species indicates that speciation in Veneridae was accompanied by some chromosome rearrangements.

Ribosomal markers have been successfully applied in species identification and, consequently, have provided valuable information regarding chromosome and genome evolution [3, 4]. However, very little is known about these sequences in the species belonging to the family Veneridae in which major rDNA sequences have only been mapped to chromosomes of six species [13, 16–18] and 5S rDNA in four species [17, 18].

The presence of major rDNA signals on just a single chromosome pair in* Venerupis corrugata*,* Ruditapes decussatus*,* Ruditapes philippinarum*,* Venus verrucosa, *and* Dosinia exoleta* is coincidental with previous results [13, 17]. Within the family Veneridae, a single major rDNA locus has been also detected in* Polititapes (Venerupis) aureus* and* P. (Tapes) rhomboides* [18], whereas* Mercenaria mercenaria* presents two major rDNA loci [16]. Therefore, our results for* V. corrugata* and* V. verrucosa *increase the number of Veneridae species showing a single major rDNA cluster to seven out of a total of eight studied. These sequences were subterminal in* P. rhomboides*,* R. philippinarum*,* V. verrucosa,* and* D. exoleta* but intercalary in* P. aureus*,* V. corrugata,* and* R. decussatus*. Furthermore, the two major rDNA clusters present in* M. mercenaria* also showed subterminal and intercalary locations [16].

The occurrence of single 5S rDNA clusters in* Venerupis corrugata* and* Venus verrucosa* matches that of* Polititapes aureus* and* P. rhomboides* [18]. On the other hand, our study also revealed the existence of two 5S rDNA clusters in* Ruditapes decussatus*,* R. philippinarum,* and* Dosinia exoleta,* which contrasts with previous findings [17, 18]. The chromosomal location of 5S rDNA clusters also showed interspecific differences. Subterminal clusters appeared in* P. aureus*,* V. corrugata*,* R. decussatus*,* P. rhomboides*,* R. philippinarum,* and* D. exoleta*, intercalary clusters in* R. decussatus* and* R. philippinarum, *and pericentromeric ones in* V. verrucosa* and* D. exoleta*.

Chromosomal mapping of both 5S and major rDNA sequences is known in a total number of 21 species of bivalves [17, 18, 24, 27–31]. Seventeen of these species show 5S and major rDNA clusters located on different chromosome pairs, and three of them also show nonlinked clusters but located on the same chromosome pair; finally,* Ruditapes decussatus* is the only one in which overlapping signals for both kinds of rDNA sequences were detected [17]. The data presented here also showed the presence of separated 5S and major rDNA signals in* Venerupis corrugata*,* Dosinia exoleta, *and* Venus verrucosa*, confirmed the absence of linkage between these sequences in* R. philippinarum* [17], and corroborated the presence of overlapping signals in* R. decussatus* [17]. Additionally, the occurrence of alternate 5S and major rDNA signals on extended chromatin fibers of* R. decussatus* suggests that these sequences are intercalated. Our results showing the existence of linkage between major and 5S rDNA sequences in* R. decussatus* but not in its congeneric species,* R. philippinarum*, or in any of the other species within the subfamily Tapetinae,* Polititapes aureus*,* Venerupis corrugata,* and* P. rhomboides,* support the suggestion that 5S and major rRNA gene linkages have been repeatedly established and lost during the evolution of eukaryotic genomes [1, 2]. These data are also in agreement with previous results reported in a wide analysis of metazoan genomes [32] in which, though 5S rDNA was found to be linked to various noncoding RNA genes in several clades, no evidence of evolutionary-conserved linkage among them was detected.

Detection of the vertebrate (T_2_AG_3_)_*n*_ repeat at chromosome ends in* Venerupis corrugata*,* Ruditapes decussatus*,* R. philippinarum,* and* Venus verrucosa* coincides with the results obtained in* Mercenaria mercenaria* [15],* Dosinia exoleta* [13],* Polititapes aureus,* and* P. rhomboides* [18]. Sequential FISH experiments using telomeric and rDNA probes on the same SC spreads also showed that in these species telomeric sequences are separated from rDNA sequences not only when the rDNA signals are intercalary but even in those species with subterminal rDNA (major or 5S) clusters. These results differ from those reported for other species and contradict the idea of Vitturi et al. [33] that in invertebrate species major rDNA clusters are closely associated with telomeric sequences.

The molecular phylogenies available for the species of Veneridae [5, 6, 8, 9] show that* Polititapes aureus*,* Venerupis corrugata*,* Ruditapes decussatus*,* P. rhomboides*, and* R. philippinarum* cluster together as members of the subfamily Tapetinae, whereas* Mercenaria mercenaria* (Chioninae),* Venus verrucosa*(Venerinae), and* Dosinia exoleta *(Dosiniinae) form separated clades. However, previous studies [6, 8] have also shown results that disagree with the classical arrangements of many currently accepted taxa [34–37]. In this regard,* R. decussatus* is closer to the species coming from the same geographic area (*P. aureus*,* V. corrugata,* and* P. rhomboides*) than to its congeneric* R. philippinarum,* while* P. aureus* is more closely related to* V. corrugata* and* R. decussatus* than to* P. rhomboides*.

The rDNA chromosomal mapping results, schematized in Figure 4 together with a simplified representation of the phylogenetic tree proposed by Chen et al. [6], indicated that the existence of single subterminal major and minor rDNA clusters on different chromosome pairs, as in* Polititapes rhomboides*, is most likely the ancestral condition. The phylogenetic relationships among the rest of the species of the subfamily Tapetinae (clade A4) could be explained, on the one hand, as a consequence of the translocation of the single major rDNA locus to an intercalary location in a common ancestor of* P. aureus*,* V. corrugata,* and* Ruditapes decussatus*, followed by an invasion of the major rDNA by some 5S rDNA sequences in* R. decussatus*. On the other hand, an independent translocation of some copies of the 5S rDNA to an intercalary location could explain the mapping data in* R. philippinarum*. In an independent event, an ancestor of* Venus verrucosa* suffered the translocation of the whole 5S rDNA cluster to an intercalary position. The presence of an additional intercalary major rDNA cluster in* Mercenaria mercenaria* and an additional centromeric 5S rDNA cluster in* Dosinia exoleta* could also be the result of this kind of event, but in this case only affecting some of the clustered copies of these genes.

In summary, this study provides further insights into the chromosomal distribution of ribosomal RNA gene clusters in species of the family Veneridae. Although the chromosomal mapping of major and minor rDNAs in these species did not clarify the phylogenetic relationship among venus clams, the striking similarity and clear differences among the chromosomal distribution of these rDNA clusters make them promising markers for the further study of chromosome evolution within Veneridae.
